# Case report: Successful treatment with spesolimab of acrodermatitis continua of Hallopeau in an older patient without *IL36RN* mutations

**DOI:** 10.3389/fimmu.2024.1440102

**Published:** 2025-01-15

**Authors:** Jianfeng Zheng, Yangfeng Ding, Yuling Shi, Xuemei Yi

**Affiliations:** Department of Dermatology, Shanghai Skin Disease Hospital, Tongji University School of Medicine, Institue of Psoriasis, Tongji University School of Medicine, Shanghai, China

**Keywords:** acrodermatitis continua of Hallopeau, pustular psoriasis, spesolimab, IL36RN, case report

## Abstract

**Background:**

Acrodermatitis continua of Hallopeau (ACH) is a rare, sterile pustular psoriasis variant refractory to many conventional treatments. The eruption typically occurs after local trauma or infection; other etiologies include neural, inflammatory, and genetic causes. Herein we reported a single case of a 64-year-old patient with ACH that was successfully treated with spesolimab for 19 weeks.

**Case summary:**

A 64-year-old Chinese male with no personal or known family history of psoriasis had recurrent episodes of redness, swelling, and pustules in the nail bed on seven fingers with progressive degeneration of the nails. The patient was monitored as to the evolution of the disease over half of a year before he referred his case to our attention. A diagnosis of ACH was made, allowing for the administration of local steroids and oral acitretin. However, after 3 months of acitretin treatment, no improvement was observed. In December 2023, this patient came to our inpatient department; his modified nail psoriasis severity index score was 32. Before starting treatment, a comprehensive set of laboratory and instrumental tests were all found to be negative. Moreover, whole-exome sequencing was performed in our patient, and it revealed no rare coding variant in *IL36RN*, *CARD14*, or *AP1S3*. Therefore, the patient was administrated with a dose of 900 mg spesolimab. After 10 days, the patient showed a significant decrease in discomfort and pain. In order to strengthen the therapeutic effect, he was given the second dose of 900 mg spesolimab after 4 weeks. After 19 weeks of spesolimab treatment, the patient’s nail lesions showed complete resolution, and no adverse effects were reported.

**Conclusion:**

The case report suggests that spesolimab may offer significant therapeutic benefits for ACH.

## Introduction

Acrodermatitis continua of Hallopeau (ACH) was first described by Henri Hallopeau in 1890. Currently, this disease, as a rare form of palmoplantar pustular psoriasis (PPP), is characterized by sterile pustules on the distal phalanges of the fingers and toes (1). Continuous inflammation results in severe finger and toe damage, onychodystrophy, and sometimes anonychia and osteolysis. The histological findings show the formation of neutrophilic pustules, degenerative changes in epidermal cells, and moderate lymphohistiocytic infiltration (2). In some cases, the onset of ACH can either precede, coincide with, or follow the development of generalized pustular psoriasis (GPP) in some cases (3, 4).

Given that ACH has a chronic and relapsing nature, achieving long-term therapeutic control becomes crucial to prevent complications. However, larger clinical studies of high methodological quality are not available due to the overall rarity of ACH. Published review articles, based on a limited number of case reports, have suggested multiple therapies including topical ointments, photochemotherapy, methotrexate, systemic retinoids, cyclosporin A, and biologics (5). However, their success rates are inconsistent (6). Therefore, there is a lack of standardized treatment guidelines for ACH.

The pathogenesis of ACH is not fully understood, but recent studies have indicated that several cytokines are involved in ACH pathogenesis. In particular, IL-36 isoforms (α, β, γ) induce transcription factors, which contribute to the unregulated secretion of inflammatory cytokines and initiate the recruitment and localization of T cells, neutrophils, and dendritic cells to the skin (7). Notably, mutations in the *IL36RN* gene, which encodes *IL36RA*, have been identified in ACH patients (8, 9). These results suggested that the IL-36 signaling pathway is a potential therapeutic target in ACH. In this case, we reported a single case of a 64-year-old patient with ACH who was successfully treated with spesolimab.

## Case report

A 64-year-old Chinese male with no personal or known family history of psoriasis had recurrent episodes of redness, swelling, and pustules in the nail bed on seven fingers with progressive degeneration of the nails. The patient was monitored as to the evolution of the disease over half of a year before he brought his case to our attention. Despite the negative findings on bacteriological/mycological examinations at another dermatological center, the patient was treated with local antibacterials and antifungals for several weeks. He had no other medical history.

Because of progressive worsening of the cutaneous clinical picture, erythema, and pustules in the bed of the nail on seven fingers, the patient visited the outpatient department of our hospital in September 2023. This patient showed obvious onychodystrophy, with frank pustules present in the bed of the nail. Fortunately, a diagnosis of ACH was made, allowing for the administration of local steroids and oral acitretin. However, after 3 months of acitretin treatment, no improvement was observed.

**Figure 1:**
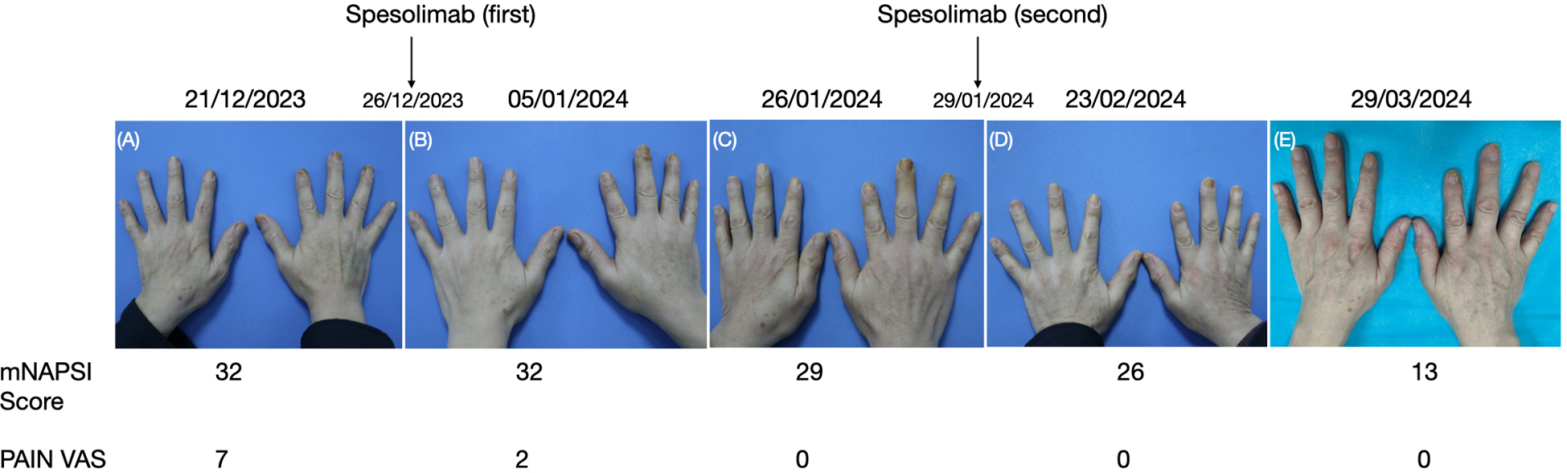
**(A–E)** Clinical presentation and the mNAPSI score and PAIN VAS before and after treatment with spesolimab.

In December 2023, this patient came to our inpatient department showing severe onychodystrophy, with frank pustules present in the bed of the nail on seven fingers (**Figure 1A**). The patient reported the maximum score on a seven-point pain visual analog scale (PAIN VAS). The patient’s modified nail psoriasis severity index (mNAPSI) score was 32. Before starting treatment, a comprehensive set of laboratory and instrumental tests were all found negative, including chest X-ray, electrocardiogram (ECG), complete blood count, complete liver profile, creatinine, autoantibodies, C-reactive protein, hepatitis B and C antibody tests, and tuberculosis assay. Moreover, whole-exome sequencing was performed in our patient, and it revealed no rare coding variant in *IL36RN*, *CARD14*, or *AP1S3*.

In the end of December 2023, the patient was administrated with a dose of 900 mg spesolimab. After 10 days, the patient showed a significant decrease in discomfort and pain (**Figure 1B**); the mNAPSI and PAIN VAS scores were 32 and 2, respectively. In order to strengthen the therapeutic effect, he was admitted to our dermatology ward again after 4 weeks. The patient’s mNAPSI and PAIN VAS scores were 29 and 0 (**Figure 1C**), respectively. Complete laboratory and instrumental tests, including ECG, complete blood count, complete liver profile, and creatinine showed, no abnormalities, so he was given the second dose of 900 mg spesolimab. The patient’s mNAPSI and PAIN VAS scores were evaluated on weeks 8 and 13 of the follow-up (**Figures 1D, E**). After 19 weeks of spesolimab treatment, the patient’s nail lesions showed complete resolution, and no adverse effects were reported.

## Discussion

ACH is a variant of pustular psoriasis with limited effective treatment options. Topical treatments (topical steroids, calcipotriol, and topical tacrolimus) are classically prescribed as first-line therapy; systemic treatments such as acitretin (ACI), methotrexate (MTX), or cyclosporin (CyA) could be efficient in some cases (2). However, the lack of treatment guidelines reflects the rarity of the disease and the challenges in disease management. In recent years, many biologics have been used for the treatment of ACH, and the results are encouraging. In Japan, K Christian et al. identified 39 patients with ACH from five university medical centers and analyzed their disease characteristics and treatment experience, which was the largest case series of patients with ACH investigating patient characteristics and treatment outcomes in a real-world setting. Among non-biologics, excellent response was noted in 21.1% (4/19) of treatment courses with MTX, followed by ACI (13.3%; 2/15). Among biologics, GUS (excellent response: 100%; 2/2), secukinumab (SEC) (excellent response: 42.9%; 3/7), and adalimumab (ADA) (excellent response: 20.0%; 2/10) were most efficacious (10).

As we know, the IL-36 signaling pathway has emerged to have a key role in the pathogenesis of GPP. Upregulation of the IL-36 cascade induces the proliferation of IL-17 and CD4+ Th17 cells, which, in turn, stimulates the expression of IL-36 and other cytokines, thereby amplifying the inflammatory response in GPP (11, 12). Furthermore, variants in many genes have been identified to be involved in the pathogenesis of ACH. N Setta-kaffetzi et al. sequenced the four *IL36RN* coding exons in nine ACH and identified recessive variants in two out of nine ACH patients, which included a 38-year-old woman carrying the p.Arg102Trp/p.Ser113Leu variants and a 77-year-old woman carrying the p.Ser113Leu variant (13).

Spesolimab is a first-in-class humanized monoclonal antibody that binds specifically to the IL-36 receptor to antagonize IL-36 signaling and inhibit the downstream activation of proinflammatory and profibrotic pathways. Spesolimab was approved by the US Food and Drug Administration (FDA) in September 2022 to treat GPP flares in adults. In the phase I study of GPP, a single dose of 10 mg/kg body weight was given to seven patients with moderate GPP (the average GPPGA score was 3). Three patients had homozygous *IL36RN* mutations, and one had a heterozygous *CARD14* mutation. By week 4, all patients had achieved clear or almost clear skin regardless of the *IL36RN* mutation’s presence (14). PF Wen et al. reported an Asian patient with a 16-year-history of GPP and ACH with marked pustulosis on the nail bed and onychodystrophy. He received the conventional systemic regimen of acitretin, cyclosporine, and biologics adalimumab and secukinumab but experienced relapse for skin lesions and refractory nail lesions. He was then treated with a single dose of spesolimab in combination with secukinumab, which resulted in skin clearance and nearly complete resolution of nail lesions over a 32-week period (4). In another case of a 9-year-old Chinese girl with a 2-year history of worsening subungual pustules, whole-exome sequencing identified two variants in the IL36RN gene. She was then treated with a dose of 450 mg spesolimab at weeks 0 and 4, which resulted in a remarkable improvement of the patient’s subungual pustules and generalized erythema over a 8-week period (15). These results suggest that spesolimab should be considered for the potential treatment of ACH.

## Conclusion

In this case, after 19 weeks of spesolimab treatment, the patient’s nail lesions showed nearly complete resolution. The obvious improvement in this patient suggests that spesolimab may be a promising therapeutic approach for ACH. However, further trials are needed to confirm the efficacy, safety, and long-term control of spesolimab in ACH patients.

## Data Availability

The original contributions presented in the study are included in the article/supplementary material, further inquiries can be directed to the corresponding authors.
